# Planctomycetes as Host-Associated Bacteria: A Perspective That Holds Promise for Their Future Isolations, by Mimicking Their Native Environmental Niches in Clinical Microbiology Laboratories

**DOI:** 10.3389/fcimb.2020.519301

**Published:** 2020-11-30

**Authors:** Odilon D. Kaboré, Sylvain Godreuil, Michel Drancourt

**Affiliations:** ^1^Aix Marseille Univ., IRD, MEPHI, IHU Méditerranée Infection, Marseille, France; ^2^Université de Montpellier UMR 1058 UMR MIVEGEC, UMR IRD 224-CNRS Inserm, Montpellier, France

**Keywords:** planctomycetes, slow-growing bacteria, host-associated extract, natural ecological niches, culture, medium, isolation

## Abstract

Traditionally recognized as environmental bacteria, Planctomycetes have just been linked recently to human pathology as opportunistic pathogens, arousing a great interest for clinical microbiologists. However, the lack of appropriate culture media limits our future investigations as no Planctomycetes have ever been isolated from patients’ specimens despite several attempts. Several Planctomycetes have no cultivable members and are only recognized by 16S rRNA gene sequence detection and analysis. The cultured representatives are slow-growing fastidious bacteria and mostly difficult to culture on synthetic media. Accordingly, the provision of environmental and nutritional conditions like those existing in the natural habitat where yet uncultured/refractory bacteria can be detected might be an option for their potential isolation. Hence, we systematically reviewed the various natural habitats of Planctomycetes, to review their nutritional requirements, the physicochemical characteristics of their natural ecological niches, current methods of cultivation of the Planctomycetes and gaps, from a perspective of collecting data in order to optimize conditions and the protocols of cultivation of these fastidious bacteria. Planctomycetes are widespread in freshwater, seawater, and terrestrial environments, essentially associated to particles or organisms like macroalgae, marine sponges, and lichens, depending on the species and metabolizable polysaccharides by their sulfatases. Most Planctomycetes grow in nutrient-poor oligotrophic environments with pH ranging from 3.4 to 11, but a few strains can also grow in quite nutrient rich media like M600/M14. Also, a seasonality variation of abundance is observed, and bloom occurs in summer-early autumn, correlating with the strong growth of algae in the marine environments. Most Planctomycetes are mesophilic, but with a few Planctomycetes being thermophilic (50°C to 60°C). Commonly added nutrients are N-acetyl-glucosamine, yeast-extracts, peptone, and some oligo and macro-elements. A biphasic host-associated extract (macroalgae, sponge extract) conjugated with a diluted basal medium should provide favorable results for the success of isolation in pure culture.

## Introduction

The history of Planctomycetes dates back to 1924 with a study of the September plankton of Lake Langymanyos (Budapest, Hungary). This study led Nador Gimesi, an Hungarian biologist to discover for the first time, an unusual planktonic microscopic organism consisting of threadlike forms which bore spherical structures floated in the eutrophic waters (Gimesi, 1924). The cryptic morphology of this organism was interpreted by Gimesi as fungal conidia and conidiophores, respectively. Therefore, this organism was described as a fungus, and named *Planctomyces bekefii* Gimesi, 1924 (Gimesi, 1924; Jenkins and Staley, 2013; Dedysh et al., 2020a). In 1935, Henrici and Johnson found morphologically similar stalked, budding microorganisms in Lake Alexander (Minnesota, USA), but as these authors were unaware of Gimesi’s previous report, they named this microorganism which they interpreted as bacteria *Blastocaulis sphaerica* (Henrici and Johnson, 1935). The authors also found spore-forming, drop-shaped, budding bacteria lacking a stalk, which they considered to be identical to *Pasteuria ramosa* Metchnikoff, 1888, a spore-forming bacterium that infects *Daphnia* species (Metchnikoff, 1888). These bacteria were later given the name *Blastobacter henricii* (Zavarzin, 1961). Since these early studies, numerous authors have reported the presence of the same or similar organisms (*Planctomyes stranskae*, *P. subulatus*, *P. ferrimorula*, *P. condensatus*, *P. guttaeformis*, *P. crassus*) from diverse habitats worldwide, including freshwater lakes, fish ponds, brackish water, aquarium water, marine sediments, forest brook, and seawater (Razumov, 1949; Ruttner, 1952; Wawrik, 1956; Zavarzin, 1960; Kahan, 1961; Skuja, 1964; Hortobágyi, 1965; Hirsch, 1968, Hirsch and Rheinheimer, 1968; Hortobágyi, 1968; OLAH et al., 1972; Hirsch, 1974; Dedysh et al., 2020a). All these organisms were described based on morphological observations with stalks that are heavily encrusted with iron. Peter Hirsch provided a new description and conclusive evidence that *Planctomyces bekefii* and *Blastocaulis sphaerica* were indistinguishable, being both bacteria rather than fungi. Indeed, Hirsch reviewed properties of both organisms, pointed to their identity, and proposed to relocate the phylum *Planctomycetes* among bacteria. Although *Planctomyces bekefii* has been described as a fungus without physiological details, priority was given to this name, of which *Blastocaulis sphaerica* must be considered a later subjective synonym (Hirsch, 1972). The initial misidentification of *Planctomyces* as a fungus, and the confusing etymology of Planctomycetes (Gr. adj. planktos wandering, floating; Gr. masc. n. mukês fungus; N.L. masc. n. Planctomyces floating fungus.), meaning “floating fungus” (Gimesi, 1924) has been conserved as such and thus explaining the current name of this phylum (*Planctomycetes*). Nearly a century (1924–2020), *Planctomyces bekefii*, the first representative described of this phylum, has remained a rare example of as-yet-uncultivated prokaryotes with validly published names and unknown identity until this 100-year-old enigma has been solved recently by Dedysh and colleagues with more precise identification (genomic characterization and biogeography), using high-performance cell sorting technology (Dedysh et al., 2020a).

Curiously, none of these authors succefully isolated any Planctomycetes in pure culture. It was not until 1973, that the first isolation of these budding, rosette-forming bacteria in a pure culture was achieved by Staley, who introduced dilute nutrient media into the practice of oligotrophic bacteria cultivation (Staley, 1973). In 1976, the first species of the phylum *Planctomycetes* was formally described on the basis of phenotypic, genetic and cultural characters (Bauld and Staley, 1976). In 1987, Carl Woese stated that Planctomycetes had a distant relationship to Chlamydiae (Woese, 1987) and Strous et al. later verified this by comparison of 49 concatenated protein sequences (Strous et al., 2006). In the same year (2006), a superphylum was designated to incorporate the phyla *Planctomycetes*, *Verrucomicrobia*, and *Chlamydiae* on the basis of comprehensive analysis of the 16S rRNAs gene (Wagner and Horn, 2006), and later by 23S rRNA gene sequences (Glöckner et al., 2010, 3) and rpo*B* (Bondoso et al., 2013). The current taxonomy includes the Planctomycetes within the PVC superphylum, which encompass *Planctomycetes*, *Verrucomicrobiae*, *Chlamydiae*, *Lentisphaerae*, *Poribacteria*, OP3, WWE2 (Cho et al., 2004; Wagner and Horn, 2006; Siegl et al., 2010; Gupta et al., 2012; Pinos et al., 2016), and a novel phylum *Saltatorellota*, recently described (Wiegand et al., 2019). According to the phylum *Planctomycetes*, it is divided into the classes *Phycisphaerae* and *Planctomycetia*. *Candidatus Brocadiae* might very well form a third class within the phylum, but no axenic cultures have been obtained from this class so far (Kartal et al., 2013). The class *Planctomycetia* was recently re-organized and is now further subdivided into the orders *Isosphaerales*, *Pirellulales*, *Planctomycetales* and *Gemmatales* (Dedysh et al., 2020b).

Some members of PVC (*Planctomycetes-Verrucomicrobia-Chlamydia*) superphylum are among the most successful human pathogens. Indeed, the pathogenicity of *Chlamydia* is no longer to be demonstrated (Belland et al., 2004; AbdelRahman and Belland, 2005). The members of the phylum *Verrucomicrobia* were found in the stool (Dubourg et al., 2013) and blood (Dubourg et al., 2017) of patients. The balance of the genus *Akkermancia* in the human gut microbiota is considered today as a potential biomarker of a healthy gut status (Belzer and De Vos, 2012) and Crohn disease (Tedjo et al., 2016). Currently, the bacteria of the *Planctomycetes* group are considered to be of increasing relevance to at least four major areas of research in microbiology: emerging models for microbial evolution, cell biology, ecology and medical interest as sources of novel bioactive compounds (antibiotics and anticancer drugs) and opportunistic pathogens (Fuerst, 1995; Jetten et al., 2001; Lindsay et al., 2001; Kuypers et al., 2003; Schmid et al., 2003; van Niftrik and Jetten, 2012; Jeske et al., 2013; Drancourt et al., 2014; Sagulenko et al., 2014; van Teeseling et al., 2015; Aghnatios and Drancourt, 2016; Graça et al., 2016; Wiegand et al., 2018; Calisto et al., 2019; Lage et al., 2019; Wiegand et al., 2020; Santos et al., 2020). Indeed, known members of the class *Planctomycetia* divide by budding and lack otherwise canonical bacterial division proteins, such as FtsZ, while the taxa *Phycisphaerae* and “*Candidatus Brocadiales*” divide by a mechanism similar to binary fission (van Niftrik et al., 2008; Jogler et al., 2012; Wiegand et al., 2020). They are capable of endocytosis and phagocytosis-like process (Lonhienne et al., 2010; Shiratori et al., 2019), sterol biosynthesis (Pearson et al., 2003; Gudde et al., 2019), nitrogen-fixation (Delmont et al., 2018), ammonia oxidation in an anaerobic double membrane (anammoxosome) which is an advantage in the treatment of polluted water (Jetten et al., 2001; van Niftrik and Jetten, 2012; Park et al., 2017a; Stultiens et al., 2020). They are also distinguished from ordinary Gram-positive and Gram negative bacteria with cell wall stabilized by a proteinaceous layer rather than a peptidoglycan layer; a characteristic shared only with the chlamydiae and mycoplasmas among the Bacteria (König et al., 1984; Liesack et al., 1986; Cayrou et al., 2012), although this is a controversial topic because recent findinds show that Planctomycetes do possess cell wall peptidoglycan (Jeske et al., 2015; van Teeseling et al., 2015). However, they are resistant to antibiotics targeting peptidoglycan (β-lactams, glycopeptides) but they are susceptible to antibiotics targeting protein synthesis (chloramphenicol, tetracyclin, doxycyclin, minocyclin, erythromycin, clindamycin) or DNA replication as Fluoroquinolone (Cayrou et al., 2010; Godinho et al., 2019). These organisms have been isolated from the soil (Ivanova et al., 2016b; Stackebrandt et al., 1993; Wang et al., 2002), hydric environments such as seawater, freshwater (Schlesner, 1994; Dedysh et al., 2020a; Wiegand et al., 2020), tap water and hospital chlorinated water systems (*Gemmata massiliana*) in proximity with patients (Aghnatios and Drancourt, 2015; Aghnatios et al., 2015). Analysis of the 16S rDNA has additionally shown the presence of *Gemmata* spp. in the gastrointestinal tract of patients with endocarditis and healthy individuals, suggesting they were members of the human digestive microbiota (Cayrou et al., 2013). More recently, using a PCR approach followed by sequencing, DNAs sequences close related to the genus *Gemmata* were detected in the blood of two febrile patients with leukemia and aplastic neutropenia (Drancourt et al., 2014). The isolation of *G. massiliana* in the water from hospital network in close proximity to these patients might supported the hypothesis of a digestive tract entry pathway by ingestion of contaminated water followed by translocation in the blood of immunocompromised patients, in whom this mechanism has been described for other bacteria belonging to the digestive tract (Tancrède and Andremont, 1985). Known as multidrug-resistant bacteria to the most commonly used antibiotics in clinical practice at usual doses (Cayrou et al., 2010; Godinho et al., 2019), and for their association recently demonstrated in humans, the great probability about the genus *Gemmata* to behave as potential opportunistic pathogens should therefore justify further investigations (Cayrou et al., 2013; Drancourt et al., 2014; Aghnatios and Drancourt, 2016).

Thus, in the practice of clinical microbiology, bacterial culture remains the gold standard for infectious disease confirmations. However, despite several attempts to isolate *Gemmata* from clinical specimens, we failed to isolate any Planctomycetes in pure culture due to the lack of suitable media and/or Planctomycetes lifestyle environmental conditions in clinical microbiology laboratories. They require a specific culture medium and long incubation times (Franzmann and Skerman, 1984; Lee et al., 2009; Aghnatios et al., 2015; Wiegand et al., 2020). Accordingly, most knowledge regarding the environmental and host-associated microbiota niche*s* of Planctomycetes organisms is derived from DNA-based studies including PCR-sequencing-based analyses and metagenomics studies (Cayrou et al., 2013; Bondoso et al., 2014; Drancourt et al., 2014; Delmont et al., 2018; Dedysh et al., 2020a), although there have been considerable advances to date, that have made it possible to isolate many species of this phylum from host-associated Planctomycetes from environmental habitats (Dedysh et al., 2020b; Jogler et al., 2020; Kallscheuer et al., 2020b; Kaushik et al., 2020; Kohn et al., 2020b; Kulichevskaya et al., 2020a; Kulichevskaya et al., 2020b; Rivas-Marin et al., 2020b; Rivas-Marin et al., 2020c; Salbreiter et al., 2020; Sandargo et al., 2020; Schubert et al., 2020; Wang et al., 2020; Wiegand et al., 2020). Hence, enormous work remains to be done to improve their culture and their isolation, in view of the enormity of 16S rRNA genes sequenced deposited in GenBank but have never yet been isolated in pure culture (uncultured Planctomycetes) up to the present day (see review Wiegand et al., 2018 for more details) because, the true knowledge of bacterial taxonomy, physiology, and pathogenicity has long time based only on the isolation of the strain in pure culture and presents several challenges for clinical microbiologists.

Also, for decades, most of the Planctomycetes studies have focused only on their diversity, distribution, and relative temporal or permanent abundance in diverse habitats (Stackebrandt et al., 1993; Borneman and Triplett, 1997; Buckley et al., 2006; Nacke et al., 2011; Steven et al., 2013; Dedysh et al., 2020a). However, research and analysis on the physicochemical compositions of the habitats of predilection of Planctomycetes remains poorly described. In this respect, we systematically review the various ecological niches of the natural habitats of the Planctomycetes in general, to have a good apprehension of their nutritional requirements, the physical and chemical factors characteristics of these natural ecological niches. Furthermore, we will emphasize the current strategies and methods of cultivation of Planctomycetes and their gaps, in the perspective to identify issues and/or opportunities in order to optimize the current cultivation conditions and protocols of these slow growing, recalcitrant and refractory bacteria to isolation. We hope that this review could help researchers choose appropriate approaches and methods to isolate new species in pure culture by applying our conclusions.

## Cultured Planctomycetes

The ﬁrst report of the isolation of a planctomycete in axenic cultures is due to the work of James T. Staley (Staley, 1973). During the last decade, several members of Planctomycetes were isolated in pure culture and described as representing new genera, namely *Schlesneria* (Kulichevskaya et al., 2007b), *Singulisphaera* (Kulichevskaya et al., 2008; Kulichevskaya et al., 2012a), *Zavarzinella* (Kulichevskaya et al., 2009), *Aquisphaera* (Bondoso et al., 2011), *Telmatocola* (Kulichevskaya et al., 2012b), *Paludisphaera* (Kulichevskaya et al., 2016; Kaushik et al., 2020), *Roseimaritima* (Bondoso et al., 2015; Kumar et al., 2020b), *Rubripirellula* (Bondoso et al., 2015; Kallscheuer et al., 2019b), *Fimbriiglobus* (Kulichevskaya et al., 2017a), *Tundrisphaera* (Kulichevskaya et al., 2017b), *Mariniblastus* (Lage et al., 2017), *Caulifigura* (Kallscheuer et al., 2020a), *Tuwongella* (Seeger et al., 2017), *Crateriforma* (Peeters et al., 2020c), *Fuerstia* (Kohn et al., 2016; Kohn et al., 2019), *Novipirellula* (Kallscheuer et al., 2019c), *Tautonia* (Kovaleva et al., 2019; Jogler et al., 2020) *Limnoglobus* (Kulichevskaya et al., 2020b), *Thalassoglobus* (Kohn et al., 2020a; Rivas-Marin et al., 2020c), *Symmachiella* (Salbreiter et al., 2020), *Gimesia* (Scheuner et al., 2014; Kumar et al., 2020a; Wang et al., 2020), *Stieleria* (Sandargo et al., 2020; Storesund et al., 2020; Surup et al., 2020), *Aureliella* (Kallscheuer et al., 2020b), *Maioricimonas* (Rivas-Marin et al., 2020b), *Alienimonas* (Boersma et al., 2019; Vitorino et al., 2020), *Lignipirellula* (Peeters et al., 2020b), *Planctopirus* (Scheuner et al., 2014; Yadav et al., 2018; Kohn et al., 2020b; Rivas-Marin et al., 2020a), *Rubinisphaera* (Scheuner et al., 2014; Kallscheuer et al., 2019a), *Lacipirellula* (Dedysh et al., 2020b), *Bremerella* (Rensink et al., 2020), *Polystyrenella* (Peeters et al., 2020a), *Rosistilla* (Waqqas et al., 2020), *Calycomorphotria* (Schubert et al., 2020), and others (Lage and Bondoso, 2011; Wiegand et al., 2020). The collection of new isolated strains has mostly increased in this year 2020, due to the remarkable work of Wiegand et al. who have isolated and characterized 79 novel planctomycetal strains, including 64 previously unknown planctomycetal species belonging to 8 known and 31 previously unknown genera (Wiegand et al., 2020). Then, 108 planctomycetal species are validly described, including 44 known species from Wiegand et al. review (Wiegand et al., 2018) and the 64 new species isolated in this year from various habitats (Wiegand et al., 2020). Most genera cited above are represented by only one or two species. The cultured strains are not at all representative of the great diversity and ubiquity that has been revealed by molecular microbial ecology techniques (Kirkpatrick et al., 2006; Penton et al., 2006; Hamersley et al., 2007; Schmid et al., 2007; Pizzetti et al., 2011b; Pollet et al., 2011; Fuchsman et al., 2012; Ivanova and Dedysh, 2012). Indeed, recent study revealed that on the 8.312 operational taxonomic units (OTUs) defined by a 99% identity threshold (full length 16S rRNA (SILVA SSU Ref NR99 database (release 128 from 07-09-2016) (Quast et al., 2013), only 0.6% of the known planctomycetal diversity on OTU level is covered by axenic cultures (Wiegand et al., 2018). Although the recent study by Wiegand and collaborators has contributed substantially with 79 functionally described and genome sequenced isolates (Wiegand et al., 2020), there are very likely even more diverse linages of Planctomycetes out there that have so far escaped detection.

## Yet Uncultured Planctomycetes

Owing to difﬁculties to obtain Planctomycetes in pure culture, the number of characterized Planctomycetes is quite limited and therefore, most studies frequently are focused on the 16S rRNA gene-based detection from various habitats (Chouari et al., 2003; Dedysh et al., 2006; Kulichevskaya et al., 2006; Shu and Jiao, 2008; Cayrou et al., 2013; Drancourt et al., 2014; Yang et al., 2016; Bondoso et al., 2017; Ivanova et al., 2018). Furthermore, the low number of Planctomycetes must be interpreted with caution, because the 16S rRNA genes of these bacteria do have mismatches to some PCR primers that are widely used in environmental diversity surveys (Pollet et al., 2011; Bondoso et al., 2013). This characteristic might lead therefore to underrepresentation of the Planctomycetes in environmental 16S rRNA libraries (see above). Less than 4% of the existing planctomycetal OTUs are partly sequenced and 99.4% still await cultivation. Metagenomics studies contribute about 250 potential planctomycetal bins which would correspond to 3% of the known diversity (Baker et al., 2015; Anantharaman et al., 2016; Kim et al., 2016; Dudek et al., 2017; Park et al., 2017b; Tully et al., 2017; Vollmers et al., 2017) while only 4 (0.05%) of the known planctomycetal genomes were obtained *via* single cell approaches clone sequences belonging to Planctomycetes have been isolated in pure culture (Quast et al., 2013; Wiegand et al., 2018). Within the classes of *Planctomycetes*, i.e., *Planctomycetia* and *Phycisphaerae*, the anammox Planctomycetes form the class *Brocadiaceae*, which has *Candidatus* status due to the lack of an axenic culture (for review see Kartal et al., 2013), although sequences that are phylogenetically affiliated with cultured heterotrophic planctomycetes were identified, the majority of the sequences belonged to several globally distributed, as-yet-uncultured *Planctomycetes* lineages (Elshahed et al., 2007). Taken together, the phylum *Planctomycetes* is heavily under-sampled and most planctomycetes remain uncultivated although their partial genomes have been detected in various environments. The current challenges of this group together represent the need to isolate new strains in pure culture to extend our knowledge of their physiological role in microbial communities, and medical interests.

## Physicochemical and Environmental Parameters Affecting Planctomycetes Geographical Distributions

Planctomycetes are widely distributed in terrestrial (Buckley et al., 2006; Ivanova et al., 2016; Stackebrandt et al., 1993; Wang et al., 2002; Slobodkina et al., 2015) and aquatic environments (Gimesi, 1924; Franzmann and Skerman, 1984; Schlesner, 1994; Glöckner et al., 2003; Pimentel-Elardo et al., 2003; Woebken et al., 2007; Sipkema et al., 2011; Webster and Taylor, 2012; Aghnatios and Drancourt, 2015) including, brackish and marine water (Wang et al., 2002; Woebken et al., 2007; Hempel et al., 2008), freshwater (Franzmann and Skerman, 1984; Wang et al., 2002; Bondoso et al., 2011; Andrei et al., 2019), and wastewater (Chouari et al., 2003; Lage et al., 2012) with diverse environmental and physicochemical conditions. Both terrestrial and aquatic habitats differing in salinity (from hypersaline to freshwater), oxygen availability (from the oxic water-column to anoxic sediments), trophic level (from oligotrophic lakes to eutrophic wastewater), and temperature (from cold-water marine snow to hot springs) (Kahan, 1961; Giovannoni et al., 1987; Kerger et al., 1988; DeLong et al., 1993; Schlesner, 1994; Vergin et al., 1998; Miskin et al., 1999; Kirkpatrick et al., 2006; Slobodkina et al., 2015; Slobodkina et al., 2016). Hence, the cosmopolitan distribution of Planctomycetes suggests a wide capacity to adapt diverse harsh environmental conditions.

First, changes in humidity and aridity affect Planctomycetes microbial communities. Indeed, the bacterial community richness and diversity are significantly positively correlated with environment with relative humidity. A study conducted to evaluate fields in drylands worldwide using DNA-sequencing approaches has found that increases in aridity reduce the diversity and abundance of soil bacteria (Maestre et al., 2015). Strongest and most significant correlations (Spearman’s rank correlation [rs] = >0.81; false-discovery rate [q] = ≤0.005) between water rate in soil and phylum relative abundance have been observed for *Acidobacteria*, *Proteobacteria*, *Planctomycetes* (*r*^2^ = 0.76), *Verrucomicrobia*, and *Euryarchaeota* (Neilson et al., 2017). This makes sense because humidity and water temperature have the greatest impact on bacterial metabolism (Scofield et al., 2015). Hence, the high rate of humidity is a strong parameter that influences the diversity and abundance of Planctomycetes. For example, in the wetlands, *Sphagnum*-dominated boreal represent one of the most extensive terrestrial environments where Planctomycetes are widespread and abundant. Northern peatlands represent a major global carbon store harboring approximately one‐third of the global reserves of soil organic carbon and these peatlands consist of acidic *Sphagnum*‐dominated ombrotrophic bogs, which are characterized by extremely low rates of plant debris decomposition (Dedysh et al., 2006; Bragina et al., 2012; Serkebaeva et al., 2013; Moore et al., 2015; Dedysh and Ivanova, 2019). Bacterial populations and environmental factors controlling polysaccharid degradation in an acidic *Sphagnum* peat in the wetland have been described in details in Obukhovskoye, Yaroslavl region, and European North Russia (Pankratov et al., 2011). The predominant populations of Planctomycetes are represented by members of the phylogenetic group of the *Isosphaera* and *Singulisphaera* (Ivanova and Dedysh, 2012; Serkebaeva et al., 2013; Ivanova et al., 2016).

Also, oxygen requirements of bacteria reflect the mechanism used by them, to satisfy their energy needs. Most members of Planctomycetes bacteria, such as *Pirellula*, *Blastopirellula*, *Rhodopirellula*, *Planctopirus*, *Gimesia*, and *Gemmata* species are chemoheterotrophic aerobes. In oxic layers of peatland sites, *Isosphaera–Singulisphaera* group are the most representatives’ genera, while anoxic peat was inhabited mostly by *Zavarzinella (Gemmata-*like*)* and *Pirellula*-like Planctomycetes. Regarding Planctomycetes related bacteria of the candidate division OP3, they are mainly detected in both oxic and anoxic peat layers (Ivanova and Dedysh, 2012). Also, some oligotrophic (*Isosphaera pallida*) and other slow-growing strains have the ability to reduce elemental sulfur to sulfide under anaerobic conditions (Fuerst, 2017). Withing the non-cyanobacterial diazotrophs inhabiting surface waters, the Proteobacteria and Planctomycetes represent the most strikingly lineages linked to nitrogen fixation in the surface ocean and impact global marine nitrogen bioavailability (Delmont et al., 2018). Among Planctomycetes, a particular group of obligately anaerobic, lithoautotrophic, the so-called “anammox” Planctomycetes, can oxidize ammonium under aerobic conditions. This process is dependent on the anammoxosome (a membrane-bound cell compartment), which might be a functional analogue of the eukaryotic mitochondrion. Anammox Planctomycetes have been found in wastewater plants, coastal marine sediments, oceanic and freshwater anoxic zones (Fuerst and Sagulenko, 2011). They have very slow growth rate (doubling times of two weeks) and their activity is inhibited by exposure to molecular oxygen even at sub ppm levels, thus it is believed that anammox bacteria are difficult to cultivate (van de Graaf et al., 1996). Futhermore, *Thermostilla marina*, a marine thermophilic anaerobic and microaerobic Planctomycete from a submarine hydrothermal vent environment can definitely use elemental sulfur as an electron acceptor generating sulfide as well as being able to respire with nitrate, using mono-, di-, or polysaccharides as electron donors (Slobodkina et al., 2016). Anaerobic metabolic abilities are widely distributed among Planctomycetes. Both cultured heterotrophic strains (*Pirellula-Rhodopirellula-Blastopirellula* clade) and yet-uncultured Planctomycetes (anaerobic ammonia-oxidizing Planctomycetes) may have the ability to use carbohydrate fermentation and sulfur reduction as possible mechanisms employed for growth and survival under anaerobic conditions (Elshahed et al., 2007; Spring et al., 2018). As an example, a Zodletone organism in the *Blastopirellula* group grows under anaerobic conditions with sulfur, probably using carbohydrate fermentation (Elshahed et al., 2007). Under acidic conditions and in low phosphate level as described in ombrotrophic wetlands, Planctomycetes use a sequential methylation to produce larger fractional abundance of mono-di-and Trimethylornithine membrane lipids to ensure their membrane stability in the micro-oxic and stress conditions (Moore et al., 2013). In summary, Planctomycetes have many capabilities to adapt under aerobic and anaerobic conditions, using diverse mechanisms.

In addition, *Planctomycetes* can inhabit in different large level of pH growth ranged from 4.2 to 11.6 (Schlesner, 1994). However, most strains are mildly acidophilic and mesophilic organisms capable of growth at pH values between pH 4.2 and 7.1 (with an optimum at pH 6.0–6.5). Some *Planctomycetes*, such has *Zavarzinella formosa*, (Kulichevskaya et al., 2009), *Telmatocola sphagniphila* (Kulichevskaya et al., 2012b), S*chlesneria*, and *Singulisphaera* are moderately acidophilic Planctomycetes growing at pH values between 4 and 7, with an optimum at pH 5–6, and they have been isolated in an acidic (pH 4.0) peat bog (Staroselsky moss, Tver region, European North Russia), (Dedysh and Kulichevskaya, 2013). The Two *Gemmata* species, are mesophilic organisms growing at pH values between 6- 8.8 (Franzmann and Skerman, 1984; Aghnatios et al., 2015). The pH is a parameter than exerts an influence on baterial community. Accordingly, two studies have reported that the pH emerges as a filter exhibiting a most important correlation with the distribution of certain soil phyla, and thus has a strong influence on the community composition as a whole alpha-proteobacteria, delta-proteobacteria, Planctomycetes, and Verrucomicrobia which were strongly correlated with soil pH (both positively and negatively correlation). The acidophilic attributes of some genera belonging to alpha-proteobacteria and *Verrucomicrobia* and the basophilic attributes of some genera belonging to Planctomycetes and delta-proteobacteria were coherent with the correlation between these taxa and pH levels between these studies (Nacke et al., 2011; Constancias et al., 2015).

### Planctomycetes Associated With Living Organisms and Their Nutritional and Intimate Relationship

In aquatic ecosystems, Planctomycetes represent up to 11% of planktonic prokaryotic communities (Neef et al., 1998; Gade et al., 2004; Bouvier and Del Giorgio, 2007; Tadonléké, 2007; Pizzetti et al., 2011a). This number is relatively low because most planctomycetes live in association with other eukaryotic organisms. Indeed, *Planctomycetes* have been found to be associated in close relationships with specific eukaryotes which enable them to expand their physiological capacities. They have been isolated from the giant tiger prawn *Penaeus monodon* (Fuerst et al., 1997), *Posidonia oceanica* seagrass (Kohn et al., 2020a), plants (Yadav et al., 2018), cyanobacterial aggregates (Kohn et al., 2016), macroalga (Bengtsson and Øvreås, 2010; Lage and Bondoso, 2012; Lage and Bondoso, 2014), marine sponges (Pimentel-Elardo et al., 2003; Kohn et al., 2020b; Wiegand et al., 2020), Sphagnum peat bog (Kulichevskaya et al., 2007b; Kulichevskaya et al., 2009), and lichens (Ivanova et al., 2016). Their sequences have been also detected in a termite *Coptotermes formosanus* gut (Shinzato et al., 2005), in an acidic Sphagnum peat bog (Dedysh et al., 2006), human oral microbiota (Takeshita et al., 2015), human gut microbiota (Cayrou et al., 2013) and from immunocompromised patient’s blood (Drancourt et al., 2014). Some of these host-associated Planctomycetes would promote the proliferation of Planctomycetes and may be act as potential sources for the isolation of new species or enriched substrata for formulation of new culture media.

### Planctomycetes Associated With Algae

A great number of published data has reported that Planctomycetes are frequently associated with the epibacterial community of several algae (Longford et al., 2007; Bengtsson and Øvreås, 2010; Burke et al., 2011; Lachnit et al., 2011; Lage and Bondoso, 2011; Lage and Bondoso, 2012; Hollants et al., 2013; Lage and Bondoso, 2014; Jogler et al., 2020; Vitorino et al., 2020). The abundant planctomycete populations on kelp surfaces and in association with other macroalga suggest that coexistence with these eukaryotes may be a key feature of many planctomycete lifestyles. Many genera and species, including *Roseimaritima ulvae*, *Rubripirellula obstinata* (Bondoso et al., 2015), *Tautonia plasticadhaerens* (Jogler et al., 2020), and others (Lachnit et al., 2011; Lage and Bondoso, 2012; Ivanova et al., 2018; Salbreiter et al., 2020; Wiegand et al., 2020) have been isolated recently from algae. Some authors speculate that the existence of a specific Planctomycetes communities associated with the algal host is likely independent of geographical variation, suggesting an symbiotic relationship (Bondoso et al., 2014). Furthermore, a specific order of *Phycisphaerales*, was proposed to accommodate the genus isolated from algae *Porphyra* sp., *Phycisphaera* (Fukunaga et al., 2009). Biofilms composed of complex communities at the surfaces of macroalga comprise a large number of bacteria, fungi and other eukaryotes embedded in extracellular polymeric organic colloids such as agarases, carrageenases, alginate lyases, dehalogenases, and antimicrobial compounds, and they constitute an interesting source of nutrients for Planctomycetes (Martin et al., 2014) as Planctomycetes are well known to contain an high number of sulfatase genes (Wegner et al., 2013), which are involved in the degradation of the sulphated polymers produced by the algae. This observation make evidence that the growth of Planctomycetes can be supported by macroalgae compounds, which provide a suitable source of nutrients that would support the growth of specific Planctomycetes. This hypothesis have been supported by growth experiments carried out with water-soluble extracts of *Ulva* sp. and *F. spiralis* (Lage and Bondoso, 2011). A specific group of OTUs were specifically associated with the type of macroalgae (Bondoso et al., 2014) and suggest the specificity of the sulfatase to metabolize diverse algal sulfated heteropolysaccharides. Also, a seasonal variation associated with the diversity and abundance of Planctomycetes have been already reported. This observation showed that Planctomycetes were highly abundant in the summer compared to winter (Dedysh et al., 2020a). These seasonal changes appeared to be related to the relative higher temperature and higher abundance of summer algae (bloom) than the differences in xenobiotic compounds in the two rivers. High levels of *Planctomycetales* during the production period have also been found in Elbe snow and in a eutrophic lake. The observation of the composition of the bacterial community of the biofilm with a continuous seasonal succession may be explained by the influence of both biotic factors such as seasonal changes in the kelp substrate and abiotic factors such as temperature. The role of Planctomycetes as degraders of sulfated polymeric carbon in the marine environment as kelps produce such substance would explained this seasonal fluctuation (Bengtsson and Øvreås, 2010; Bengtsson et al., 2010). In general, Planctomycetes are more abundant in samples collected in summer and earlier-autumn than in samples collected in winter and spring. Some authors found that samples collected in June contained the highest cell numbers of cyanobacteria and microscopic algae (Dedysh et al., 2020a), these fluctuations seemed related to the algae, cyanobacteria, or diatom bloom of, which constitute a nutrient source for Planctomycetes. In addition, the successive blooms of, Dinoflagellates and Pennales might provide different types of substrates which support the growth of specialized clades of Planctomycetes (Pizzetti et al., 2011a; Lage and Bondoso, 2014). In this regard, many strains of Planctomycetes have been isolated when sampling takes place in summer. Marine macroalga have emerged as significant habitats for Planctomycetes and sources of inoculum for isolation (Bengtsson and Øvreås, 2010; Lage and Bondoso, 2014; Jogler et al., 2020; Salbreiter et al., 2020; Wiegand et al., 2020). More than 140 planctomycetes from the bioﬁlm community of macroalgae have been isolated by Lage and colleagues (Lage and Bondoso, 2011). In addition, Planctomycetes associated with algae can secrete compounds that might serve as trigger to stimulate the secondary metabolite production in Planctomycetes for drug development (Jeske et al., 2013). Despite increasing knowledge of the successful association of Planctomycetes and macroalgae, considerable effort is required to fully understand this interaction (bacterial attachment *via* Planctomycetes holdfast or stalk and/or *via* chemotaxis or a symbiotic relationship). An identification and purification of these polysaccharides secreted by algae could be a research track to test them individually and serve as growth factors to enrich new culture media of Planctomycetes.

### Planctomycetes Associated With Sphagnum and Lichens

Planctomycetes can also be found in association with lichens (Ivanova et al., 2016) and *Sphagnum* (*Sphagnaceae*) peat bogs (Dedysh et al., 2006; Kulichevskaya et al., 2006; Kulichevskaya et al., 2007b; Pankratov et al., 2011). Authors reported that Planctomycetes make up an important part of the bacterial population responsible for *Sphagnum* decomposition, accounting for up to 14% of total bacterial cells (Kulichevskaya et al., 2006; Kulichevskaya et al., 2007a; Ivanova and Dedysh, 2012). Several peat-inhabiting Planctomycetes representatives, including the genera, *Schlesneria*, *Singulisphaera*, *Telmatocola*, *Paludisphaera*, *Tundrisphaera* (Kulichevskaya et al., 2007b; Kulichevskaya et al., 2008; Kulichevskaya et al., 2009; Kulichevskaya et al., 2012a; Kulichevskaya et al., 2012b; Kulichevskaya et al., 2017b; Dummy, 2017), and most of *Gemmata* like-related Planctomycetes (*Zavarzinella formosa*, *Telmatocola sphagniphila*, *Fimbriiglobus ruber*) have been recovered from *Sphagnum* moss-dominated wetlands (Kulichevskaya et al., 2007b; Kulichevskaya et al., 2008; Kulichevskaya et al., 2012b; Ivanova and Dedysh, 2012; Kulichevskaya et al., 2015). Northern peatlands represent a significant global carbon store and commonly originate from *Sphagnum* moss-dominated wetlands. These ombrotrophic ecosystems are rain fed, resulting in nutrient-poor and acidic conditions. Members of the bacterial phylum *Planctomycetes* have been found to be very highly abundant and appear to play an important role in the decomposition of *Sphagnum*-derived litter in these ecosystems. (Kivinen and Pakarinen, 1981; Gorham, 1991). Growth experiments showed that peat-inhabiting Planctomycetes have the ability to catabolize a great number of heteropolysaccharides belonging to *Sphagnum* peat, as the addition of *Sphagnum* peat resulted in the relative abundance of Planctomycetes compared to the total microbial community (Kulichevskaya et al., 2007b; Pankratov et al., 2011; Kulichevskaya et al., 2012b). Also, in a recent experiment, performed to compare the microbial communities of two lichen-dominated ecosystems of the sub-arctic zone of northwestern Siberia, that is a forested tundra soil and a shallow acidic peatland, authors have shown that soil and peat layers just beneath the lichen cover were abundantly colonized by Planctomycetes, ranged from 2.2 to 2.7 × 10^7^ cells/gram of wet weight, using molecular tools. In addition, authors noticed that lichen-associated assemblages of Planctomycetes displayed unexpectedly high diversity, with a total of 1.723 OTUs determined at 97% sequence identity. Uncultivated members of the *Planctomycetaceae* (53%–71% of total *Planctomycetes*-like reads), formed the most abundant populations of forested tundra soil while sequences affiliated with the *Phycisphaera*-related group (order *Tepidisphaerales*) were most abundant in peat (28%–51% of total reads). From both habitats, representatives of the *Isosphaera*–*Singulisphaera* group (14%–28% of total reads), *Gemmata* (1%–4%), and *Planctopirus*–*Rubinisphaera* (1%–3%) were represented (Ivanova et al., 2016). Finally, this observation suggests that both *Sphagnum* and lichen are a potential sources of growth factors that would revolutionize the culture of yet uncultured Planctomycetes. Considerable effort is required to better understand these relationships in order to identify and purify these presumably growth factors secreted by the Sphagnum/lichens to enrich new Planctomycetes culture media.

### Planctomycetes Associated With Other Bacterial Communities

In nature, no organism exists in isolation and biological interactions are inevitable. Most DNAs of the Planctomycetes, including those of the genus *Gemmata* are constantly detected is association with some other bacterial phyla. In general, the bacterial community structures in the most studies, revealed a great association between *Alphaproteobacteria* and *Planctomycetes*, followed by others such as *Bacteroidetes*, *Gemmatimonadetes*, and *Verrucomicrobia* (Wang et al., 2011; Kim et al., 2014; Miyashita, 2015; Delmont et al., 2018), suggesting symbiotic co-operation with these bacteria. In ecology, these interactions can either be intraspecific, involving only members of the same species, or interspecific, involving one or more different species. Thus, in both aquatic, terrestrial environments and human gut, Planctomycetes are commonly associated with other bacteria-eukaryote where they form multispecies assemblages. While these assemblages can form on basically with all bacteria, Planctomycetes associated with other bacterial clades were found to play important roles in supporting the growth of Planctomycetes by providing them with nutrients. Major bacterial groups found in these associations are, for example, *Proteobacteria*, *Bacteroidetes*, and *Verrucomicrobia* which are dependent on each other. Thus, possible explanations for the resistance of yet uncultured Planctomycetes in purity *in vitro* include: unmet fastidious growth requirements; inhibition by environmental conditions (pH, temperature, attachment) or chemical factors produced by neighboring organisms bacteria in mixed cultures; or conversely, dependence on interactions with other organism in the natural environment, without which they cannot survive in isolation. Some Planctomycetes, with metabolic pathways lacking in the necessary genetic material to encode for essential nutrients, frequently rely on close symbiotic relationships with other bacteria for survival and may therefore be recalcitrant to cultivation in purity. Then, the absence of some complex conditions of cultivation in clinical microbiology laboratories has contributed to numerous isolation failures of fastidious species of bacteria that were considered “unculturable”. To this end, providing environmental and/or nutritional conditions like those in the natural habitat of these bacteria could be an interesting option for the improvement of culture and the success of isolation. As an example, we have shown with genomic studies that *Gemmata* spp. lack a complete set of genes involved in iron acquisition, suggesting an iron-based cooperation between *Gemmata* spp. organisms and *Proteobacteria* prototype *Escherichia coli*. Testing co-culture of *Gemmata* spp. with *E. coli* filtrate, we showed that the number of both *G. obscuriglobus* and *Gemmata massiliana* colonies were significantly higher on basic medium supplemented with *E. coli* filtrate than on the standard medium (p < 0.0001) (Kaboré et al., 2019a). Hence, cooperating groups might exist between uncultured *Planctomycetes* and *Proteobacteria* like *E. coli* for siderophore and iron acquisition, which causes the interests of these individuals to be associated with those of the group and other such as *Verrucomicrobia* and *Actinobacteria* (D’Onofrio et al., 2010).

### Planctomycetes Associated With Natural Sponges

In the marine environments, Planctomycetes are often associated with sponge surfaces where they form multispecies assemblages which are called biofilms. While these assemblages can form on basically every surface, bacteria associated with aquatic eukaryotic phototrophs, such as macroalgae or seagrass and marine sponges (Pimentel-Elardo et al., 2003; Kohn et al., 2020b; Wiegand et al., 2020), were found to play important roles in supporting the growth of their hosts while they are the same time live off the nutrients provided by the host organism. A great number of studies have revealed that Planctomycetes are commonly associated with marine sponges (Wilkinson and Fay, 1979; Pimentel-Elardo et al., 2003; Zhu et al., 2008; Mohamed et al., 2010; Ouyang et al., 2010; Sipkema et al., 2011; Webster and Taylor, 2012; Costa et al., 2013; Izumi et al., 2013; Kohn et al., 2020b; Wiegand et al., 2020). Sponges host diverse microorganisms that can constitute up to 60% of the total sponge biomass (Vacelet and Donadey, 1977; Wilkinson, 1978; Hentschel et al., 2006; Taylor et al., 2007). Most marine sponges establish a persistent association with a wide array of phylogenetically and physiologically diverse microbes. Sponge-microbe associations involve a diverse range of heterotrophic bacteria, including Planctomycetes, Verrucomicrobia, cyanobacteria, facultative anaerobes, unicellular algae, and archaea (Pimentel-Elardo et al., 2003; Hoffmann et al., 2005; Scheuermayer, 2006; Schmitt et al., 2012). Symbiotic relationships between sponges and microorganisms contribute to the sponges’ health and nutrition. These relationships can involve more than one partner and can vary from mutualism to commensalism to parasitism. In contrast, sponges may offer nourishment and protection to their symbionts (Bultel-Poncé et al., 1999), and the symbionts may benefit the nutrition of their host by translocation of metabolites through, for example, nitrogen fixation, nitrification, and photosynthesis (Wilkinson and Fay, 1979; Wilkinson and Garrone, 1980; Schläppy et al., 2010; Ribes et al., 2012). On the surface of marine sponges, a large diversity of planctomycetes has been observed among bacteria that are constantly resident in the microbial biofilms of the marine environment. These interactions between the two organisms are either promoted by bacterial attachment *via* Planctomycetes holdfasts and/or *via* chemotaxis or a symbiotic relationship. Indeed, culture-dependent and independent methods have revealed the existence of many cultured and uncultivated species in the epibacterial communities of the marine sponge. Several factors have been implicated in the colonization of sponge surfaces by Planctomycetes, including the adhesion factors present on the surface of certain species of Planctomycetes (Stalks, holfasts, frimbriae) that favor their attachment to marine sponges. On the other hand, these associations could be explained by the fact that the sponges which secrete various unknown molecules, or sulphated polysaccharides (which are the substrate of the abundant sulfatases produced by the Planctomycetes) (Wegner et al., 2013) *via* the algae hosted by the sponge, or molecules such as siderophores secreted by Proteobacteria associated with biofilms. There appears to be some specificity between certain planctomycete species that frequently associate with sponges (Pimentel-Elardo et al., 2003). The nature of this association could also be related to the chemical nature of the sulphated polysaccharides produced by each alga associated with sponge in the biofilms. In addition, since Planctomycetes are resistant to many antibiotics (Cayrou et al., 2010), this property would allow them to resist the bactericidal action of several antimicrobial compounds produced by sponges (Doshi et al., 2011) against other bacteria associated in these biofilms community. This selects Planctomycetes to the detriment of other bacterial species. Accordingly, we have conducted a study on this interaction and observed that the heat-aqueous extract and small tissues of the marine sponges *Spongia* sp. skeletons were able to improve the growth of *Gemmata massiliana* and *Gemmata obscuriglobus* through mechanical and growth factors mechanisms (Kaboré et al., 2019b). In conclusion, an identification and purification of these molecules should be carried out in the perspective to enrich Planctomycetes culture media.

### Planctomycetes Associated With Human Microbiota

At our knowledge, until now, there are no published data that reports the isolation of Planctomycetes in pure culture from human microbiota. However, Planctomycetes organisms are part of the human microbiota and the genus *Gemmata* spp. is the most commonly (50%) Planctomycetes-associated with human as their partial sequence of 16S rRNA gene have been found in 6/12 individuals (De Hertogh et al., 2006; Maldonado-Contreras et al., 2011; Cayrou et al., 2013), and recently, their sequences have been detected in the blood collected from two immunocompromised aplastic patient (Drancourt et al., 2014), although we failed to isolate any Planctomycetes in pure culture. Cayrou et al. (2010) observed that when patients received peptidoglycan inhibitor antibiotics they had the high prevalence of Planctomycetes, which data are in agreement with the demonstrated resistance of peptidoglycan-less Planctomycetes organisms to such antibiotics (Liesack et al., 1986; Cayrou et al., 2010; Godinho et al., 2019). Similar opportunistic behaviors have been also reported with the neibouring clade-*Verrucomicrobia* from PVC superphylum (Dubourg et al., 2013; Dubourg et al., 2017). Originally known as typical environmental bacteria and neglected in clinical microbiology laboratories, recent year, Planctomycetes have gain many interests (Aghnatios and Drancourt, 2016). However, these data reported in humans so far could only represented the tip of the iceberg. Indeed, their DNA remain problematic as the 16S rRNA gene PCR primers routinely used to detect bacteria in microbiology laboratories failed to detect some Planctomycetes organisms (Vergin et al., 1998). In addition, Christen and colleagues on the basis of the detection on rpo*B* gene argue that Planctomycetes escape standard clinical diagnostics and certain methods were suggested to overcome this limitation (Bondoso et al., 2013; Christen et al., 2018). Nowadays, the diversity and variation investigations (antibiotherapy) of human microbiota presents several challenges to microbiologists. Originally dominated by culture-dependent methods for exploring this ecosystem, the advent of molecular tools has revolutionized our ability to investigate these relationships. Microbial culturomic, a concept based on a use of several culture conditions with identification by MALDI-TOF followed by the genome sequencing of the new species cultured had allowed a complementarity with metagenomics but Planctomycetes have been neglected by most human microbiota studies using culturomic approaches. This is due in large part to the fact that they grow very slowly compared to ordinary bacteria (such as *E. coli*) and their low nutrient requirement in the culture media where they usually grow compared to enriched media commonly used in clinical microbiology. In the future, studies using complementary methods from a broad range of both culture-based and molecular tools will increase our knowledge of the repertoire of this complex ecosystem and host-Planctomycetes mutualism. So far, it remains enigmatic whether Planctomycetes play an active role in human pathogenesis and today, there is no conclusive statistically significant evidence, that members of the phylum Planctomycetes are responsible for any kind of known disease.

## Gaps in the Current Media for Planctomycetes Isolation

A great technological accomplishment using several culture methods and media formulations leading to the isolation of a great number of Planctomycetes isolates from various habitats were subsequently performed in 1994. These breakthrough were especially due to Dr. Heinz Schlesner’s work (Schlesner, 1994), and recently by (Wiegand et al., 2020). Currently, the formulation and development of many Planctomycetes isolation media existing varied with respect to the Planctomycetes species targated from diverse habitats with a wide range of salinity (fresh to hypersaline water), pH (4.2–11.6), trophic levels (oligotrophic vs. eutrophic) and few differences in the composition and concentrations used. The culture media that have allowed the isolation of current Planctomycetes cultured organisms employ usually chemoheterotrophs low nutrient-media containing peptone (0.05% or less), yeast extract (0.1 g/L or less), glucose (0.025%) plus trace elements (MgSO_4_, CaCO_3_, FeSO_4_). The introduction of N-acetylglucosamine (1g/L or less), the monomere of chitin, which constitutes both a carbonaceous and nitrogenous source for Planctomycetes metabolism has allowed Schlesner and Wiegand et *al*. to the isolation of numerous strains (Schlesner, 1994; Lage and Bondoso, 2012; Wiegand et al., 2020). However, some Planctomycetes can grow in quite nutrient rich media like M600/M14 medium (Lage and Bondoso, 2011; Graça et al., 2016). Most authors commonly use sometimes the addition of complex vitamins named Staley vitamin solution (see medium 600 from DSMZ), required by some members of the Planctomycetes to the isolation media. Also, some micronutrients (Hutner’s salts (see medium 590) and macronutrients (see medium 600) are used (Schlesner, 1994; van Niftrik and Devos, 2017; Wiegand et al., 2020). In the literature, most culture media used are Staley’s maintenance medium (see medium 629) as described from https://www.dsmz.de/microorganisms), the M1N, M31, M600/M14 (Kulichevskaya et al., 2009; van Niftrik and Devos, 2017), PYGV (Peptone, Yeast extract, Glucose supplemented with 20 ml Hutner’s basal salts and Vitamin solution) (Staley, 1968), and Caulobacter medium (Christen et al., 2018; Kaboré et al., 2018). In all these previously described media, in general, Planctomycetes are comparatively slow growing organisms with low demand for carbon and nitrogen sources. This makes them difficult to isolate in routine clinical bacteriology media, because they easily outgrown by bacteria with faster growth rates (such as *E. coli*, doubling time: 20 min). Indeed, in a non-selective medium, competition between Planctomycetes and fast-growing microorganisms hampers their isolation by agar invasion and depletion of nutrients. However, antibiotics targeting the petidoglycan biosynthesis, such as β-lactam antibiotics (1 mg/L penicillin, 200 mg·ml^−1^ ampicillin and, 100 mg/L imipenem), aminoglycosides (1,000 mg·ml^−1^ streptomycin), and glycopeptides (40 mg/L vancomycin), are commonly added to the growth media and solve this problem with the selective isolation of a large collection of culturable Planctomycetes from polymicrobial samples (Wang et al., 2002; Lage and Bondoso, 2012; Lage et al., 2012; Lage and Bondoso, 2014; Wiegand et al., 2020). Besides the overgrowth of rapid growing bacteria, another problem, commonly faced when isolating bacteria from environmental samples, is the rapid and invasive growth of fungi. The bottleneck in these cases are usually solved by addition to the growth media of cycloheximide or amphotericin B (Schlesner, 1994; Wang et al., 2002; Winkelmann and Harder, 2009; Cayrou et al., 2010). However, these antifungal compounds have not always proven to be effective and fungicides like pevaryl (econazole nitrate; 1%) and benlate (benomyl, or methyl l-(butylcarbamoyl)-2-benzimidazole carbamate;4 mg·ml^−1^) appear to be more adequate in inhibiting fungal growth (Lage and Bondoso, 2011). Futhermore, when the targated strains belong from marine sample, the authors commonly use filtired marine water or artificial seawater in order to complement the medium and to reach the bacterial natural environment (Schlesner, 1994; Mu et al., 2020; Wiegand et al., 2020). This was especially relevant for marine aquatic Planctomycetes. In contrast, when the targated strains is a freshwater Planctomycetes, tap filtired waters are used or soil extracts for soil strains (Wang et al., 2002; Aghnatios et al., 2015). For exemple, the culture medium used to isolate *G. obscuriglobus* (freshwater bacterium) consisted of 2.0 µm filtered lake water solidified with 1.5% agar. Micro-colonies were then subcultured using a microloop and a microforge (device and technique described by Skerman) to a new plate to boost their growth (Skerman, 1968; Franzmann and Skerman, 1984). Similar strategy has been used to isolate and culture *G. massiliana* from Hospital network water supplemented with the basic culture medium with the appropriate nutrients (Aghnatios et al., 2015). According to the salinity, temperature, and pH growth, for *Gemmata* spp., freshwater bacteria, salinity must be <0.6% for *G. obscuriglobus* and 1.5% for *G. massiliana*, glucose concentration ≤0.1% and pH growth range at (7.8–8.8). For *Gemmata*-like Planctomycetes, salinity must be <0.6% (*Zavarzinella formosa*), glucose concentration ≤0.05% (*Zavarzinella formosa*), ≤0.025% and pH growth range (4.0–7.0), temperature range, 6°C–30°C with an optimum at 20°C–26°C. Both *Gemmata* spp. and *Gemmata*-like should be required a long time of incubation to be isolated. The difficulties related to the culture of Planctomycetes is due to their generation time relatively very long (e.g., *G. obscuriglobus* exhibit a 13-h generation time, *Gimesia maris* 13–100 h depending on medium, and the anammox Planctomycetes with typical generation times of more 2 weeks (Fuerst, 1995; Lee et al., 2009; Fuerst, 2017). In addition, the need for attachment to support to produce a mobile bud (Fuerst, 2004) in a liquid medium would make the task difficult on ordinary solid agar. Also, the premature drying of agar plates in petri dishes is a one limit for long time incubation. Hence, to circumvent these isolation bottlenecks, news culture media formulation, novel approaches and technical manipulations are needed to recovery news strains from various habitats, including human blood which are very important for us.

## Discussion and Perspectives of Methods and Sources for the Enrichment and Isolation of Planctomycetes in Pure Culture

Unless we bring proof by culture in several patients, it remained enigmatic whether Planctomycetes play an active role in human pathogenesis. Moreover, knowledge on certain aspect of the biology of microorganisms cannot be reached unless the organisms are available in culture, but the problem of uncultivability remains a major challenge. Here, we reasoned that uncultivable recalcitrant microorganisms might grow in pure culture if provided with the chemical components of their natural environment. Hence, after going through the literature data, we found common points about the isolation, culture methods of the Planctomycetes strains that have been isolated by the authors, and certain perspective to help both clinical and environmental microbiologists to improve Planctomycetes cultivability. Thus, since the introduction of low nutrient-media in Planctomycetes culture by Staley (Staley, 1973), a great technological accomplishments, several culture methods and media formulations for the isolation of planctomycetes were subsequently performed by others (Schlesner, 1994; Lage and Bondoso, 2012; Wiegand et al., 2020). Since most budding Planctomycetes lived in the attached state, the need of cell to attach to a solid support to ensure their reproduction (Henrici and Johnson, 1935) in the natural environment, the task would be difficult on ordinary solid agar used routinely in clinical microbiology laboratories for isolation. Hence, for the success of isolation, several enrichment techniques should be tried, and some enrichment experiments should take several months. The technology of prior enrichment on cover glass and the so-called “Petri-dish method” (Hirsch et al., 1977) have allowed Schlesner’s to recover many isolates (257 strains) from various habitats in pure culture after several months, using diluted media (Schlesner, 1994). The petri dish technique, taking a great advantage of attachment of Planctomycetes to glass surfaces, was especially important for increasing markedly the numbers of Planctomycetes for further isolation (Schlesner, 1994). Indeed, allowing samples with or without addition of low concentrations of substrata to stand for a long time or generally employing nutrient-poor media were most successful. Dilute media were preferred and taking a great advantage since Planctomycetes are overgrown by faster growing bacteria (Hirsch and Müller, 1985). Also, in a polymicrobial sample such as stool, competition between Planctomycetes and fast-growing microorganisms occurs and hampers their isolation by agar invasion and depletes nutrients. However, the fact that Planctomycetes are peptidoglycan-less involves the use of antibiotics (see above) targeting the peptidoglycan to solve this problem and allow Planctomycetes to form colonies on the isolation plates (Cayrou et al., 2010; Godinho et al., 2019). Besides the overgrowth of rapid growing bacteria, another problem, commonly faced when isolating bacteria from clinical samples, is the rapid and invasive growth of fungi (*Aspergillus*, personal data) despite the addition of amphotericin B (Christen et al., 2018). Some authors commonly use cycloheximide to inhibit fungal growth (Schlesner, 1994; Wang et al., 2002; Winkelmann and Harder, 2009) but some others prefer fungicides like pevaryl (1%) and benlate (4 mg·ml^−1^) for its effectiveness (Lage and Bondoso, 2011; Lage and Bondoso, 2012). According to strains isolated so far, the most common technique of isolating Planctomycetes still consists of taking the natural habitat (depending on whether the habitat is fresh water, marine, brackish, soil extract, or tap water) where Planctomycetes have been detected by molecular tools, to then transport the sample (liquid-solid) of this natural habitat to the laboratory which will serve as a basis for the preparation of enrichment and isolation media. Once in the laboratory, the sample of the natural habitat will be filtered (0.22 μm) and this filtrate will serve as a basis for the preparation of enrichment and isolation media (Franzmann and Skerman, 1984; Schlesner, 1994; Lage and Bondoso, 2012; Aghnatios and Drancourt, 2015; Wiegand et al., 2020). Then, the resulted sterile filtrate usually supplemented by the following components (gram per liter of sterile filtrate): peptone (0.05% or 0.025%), yeast extract (0.1 g or 0.025%), glucose as carbon source, N-acetylglucosamine (1g/L, C and N_2_ sources) and Staley vitamins solution (see media section above for more details). According to vitamin addition, only vitamin B12 (cyanocobalamin) and Vitamin B6 (biotin), used together have proved their effectiveness to potentiate the growth of *Gemmata obscuriglobus* in recent study and remain sufficient to restore colony growth to comparable rates as other commonly used media (Staley, 1968; Schlesner, 1994; Mishek et al., 2018). For the addition of micro- and macronutrients belonging from the Hutner’s basal salts, (component of Staley’medium) some authors state that it produced favorable results (Cohen-Bazire et al., 1957; Schlesner, 1994), however, for the fresh water bacteria such *Gemmata massiliana* and *Gemmata obscuriglobus* (salinity must be <0.6%), its addition does not proved its effectiveness compared to basic Caulobacter medium (2 g/L Bacto peptone,1 g/L yeast extract and 0.2 g/L MgSO4 heptahydrate, DSMZ Medium 595) (Poindexter, 1964; Christen et al., 2018; Kaboré et al., 2018). In order to improve the Planctomycetes isolation from clinical and environmental laboratory, we recommended, on the basis of the literature data that: i) sphagum and lichen gained a great attention to be help researchers to isolate *Gemmata*-like Planctomycetes and other yet-uncultured Planctomycetes from clinical samples as most *Gemmata*-like have been isolated in these natural habitats (*Zavarzinella Formosa*, *Telmocola sphagniphila*) from Sphagum-peat bog and lichens (Kulichevskaya et al., 2008; Kulichevskaya et al., 2009; Kulichevskaya et al., 2012b; Kulichevskaya et al., 2017a; Kulichevskaya et al., 2017b). ii) Furthermore, many Planctomycetes colonies have been recovered from the surface of small portions of macroalgae and algae water-extracts have proved that the growth of Planctomycetes can be supported by macroalgae compounds (Lage and Bondoso, 2011; Wiegand et al., 2020). This hypothesis has been supported by the nutritional role of macroalgae for Planctomycetes (Lage and Bondoso, 2011; Lage and Bondoso, 2012). Indeed, water-soluble extracts (used as macroalgal macerated) of *Ulva* sp. and *F. spiralis* have proved their effectiveness to improve the growth of some Planctomycetes as certain Planctomycetes possess many sulfatases to metabolize the sulfated heteropolysaccharides produced by algae. This explain their strong association with algal species and has allowed Lage and colleagues to obtain a large collection of culturable Planctomycetes, essentially from the surface of macroalgae (Lage and Bondoso, 2014). iii) In addition, marine sponge heat- aqueous extracts sterilized by autoclaving should be constitute a potential novel source of growth factor and basic micro-and macronutrient belonging from natural environment to revolutionize Planctomycetes culture. Prior to plated the inoculum into agar plate, the sample should be enriched at 3 or 4 days and more [2–4 month, see (Schlesner, 1994)] until a turbidity occurred. This has been supported by previous studies (Pimentel-Elardo et al., 2003; Winkelmann et al., 2010; Webster and Taylor, 2012). Better, the liquid culture should be associated with a solid phase for stalked or budding bacteria attachment during enrichment period. The highest number of cells was usually found on the walls of the vessels and many mature cells are attached to surfaces (sponge, sphagnum, and macroalgae). As an example, after obtaining the media, a portion of the sample should be concentrated (e.g., 2 g of wet peat suspended in 10 ml of sterile water and treated in a laboratory stomacher at 240 rpm for 5 min has allowed to isolate *Telmatocola sphagniphila*) to obtain a small volume of bacteria to be inoculated (Kulichevskaya et al., 2012b). Then, the resulting suspension can be used to inoculate serum bottles containing 90 ml of sterile dilute medium described above (after pH adjustment as close as possible to the natural habitat). The enrichment media should be incubated in the dark or light at room temperature (mesophilic) for at least 2–4 weeks or even 2–7 months (Schlesner, 1994). Method of coverslips or petri-dish described above will provide support of attachment, necessary for the bacteria of interest that are Planctomycetes for their attachment. Twenty-microliter aliquots of the resulting enrichment cultures could be plated onto solid medium containing appropriate antibiotic and fungicide described above. The microbial cells that will develop in contact with the glass can be observed microscopically to detect the presence of cells with a morphology similar to those of Planctomycetes in the enrichment broth before seeding on a solid version of the same liquid medium (agar addition). Some plates could be incubated in gastight jars containing 5% CO2 (v/v) in air. Humidification of petri dishes should be controlled to prevent premature drying of agar plates. This humidification could be ensured by introducing the petri dishes in a jar with Kleenex paper soaked in water. Colonies that developed on plates must be screened microscopically for the presence of cells with Planctomycetes-like morphologies. The selected cell material should be re-streaked onto the same medium and this procedure must be repeated until the target microorganism was obtained in a pure culture. Finally, as an alternative, another isolation technique which are relevant and commonly used is the micromanipulation using the methods and apparatus described by (Skerman, 1968) to separate a Planctomycetes isolate from a co-cultured of non Planctomycetes isolate after the failure of conventional sub culturing method.

## Conclusions

In microbiological laboratories, most yet uncultured Planctomycetes lack the environmental conditions like those like “*Chlamydia* or virus are need host to express it life”. Accordingly, several factors have been implicated in the colonization of host organisms by Planctomycetes, including the adhesion factors present on the surface of certain species of Planctomycetes (Stalks, holfasts, frimbriae) that favor their attachment to host (marine sponges, macroalgae) to ensure their budding and rapid growing. On the other hand, these associations could be explained by the fact that these host organisms secrete various unknown molecules, or sulphated polysaccharides (which may be the substrates for the abundant sulfatases produced by the Planctomycetes) *via* the algae hosted by the sponge, lichen, moss, or molecules such as siderophores secreted by Proteobacteria associated with biofilms for these Planctomycetes nutrition. Hence, the need to recovery yet uncultured Planctomycetes should take these process account to complemented current culture media with other bacterial filtrates. New isolation approaches by combining a new combination of new media formulation (including sponge extracts, macroalgae macerate, lichens and mosses extracts, *E. coli* filtrate and iron, vitamin B12 and vitamin B6, and *N*-acetylglucosamine), culture approaches (host-soluble extracts and host-tissues as solid support to prepare an ecological biphasic medium), antibiotics, fungicides, and the use of low nutrient media should be essential for successful isolations. We hope that our review will help researchers achieve this goal.

## Author Contributions

OK wrote the initial manuscript. SG and MD proposed the review subject. OK, SG, and MD drafted the manuscript. All authors contributed to the article and approved the submitted version.

## Conflict of Interest

The authors declare that the research was conducted in the absence of any commercial or financial relationships that could be construed as a potential conflict of interest.
